# Depression, Anxiety and Criminal Behaviour Between Ages 32 and 48: A Propensity Score Matching Analysis From the Cambridge Study in Delinquent Development

**DOI:** 10.1002/cbm.70036

**Published:** 2026-05-06

**Authors:** Kim Reising, Maria M. Ttofi

**Affiliations:** ^1^ School of Social and Political Sciences University of Lincoln Lincoln UK; ^2^ Institute of Criminology University of Cambridge Cambridge UK

**Keywords:** anxiety, Cambridge Study in Delinquent Development (CSDD), criminal behaviour, depression, longitudinal study, propensity score matching

## Abstract

**Background:**

Depression, anxiety and criminal behaviour are often correlated, but the direction and nature of these associations remain contested.

**Aims:**

To investigate the temporal relationships between depression and/or anxiety and criminal behaviour at age 32 and depression and/or anxiety and criminal behaviour at age 48.

**Methods:**

Using prospective, longitudinal data from the Cambridge Study in Delinquent Development, we applied propensity score matching to estimate the associations between (a) depression and/or anxiety at age 32 and criminal convictions at age 48 and (b) criminal convictions at age 32 and mental health problems at age 48. Propensity score matching (PSM) was used to adjust for a wide range of childhood and adolescent risk factors for offending or illness.

**Results:**

Overall, a significant association between depression and anxiety and much later offending was evident, but after allowing for childhood adversities by propensity score matching, these relationships disappeared, suggesting that they are best explained by the common antecedent of childhood adversities. There was no relationship between any offending up to age 32 and depression or anxiety measured at age 48.

**Conclusions:**

Although our findings were limited by the very large time gap between measures of mental health and offending, it is clear that depression and anxiety may be important in the pathway to crime, but that this association is largely accounted for by shared early‐life risk factors. Our findings also, however, caution against assuming any direct relationship between these symptoms and crime, regardless of direction. Recognising early developmental vulnerabilities and intervening appropriately may be the most effective way of preventing later ill health and criminal behaviour.

## 1 Introduction

Mental disorder and criminal behaviour are pressing problems worldwide, reflected in the United Nations (UN) Sustainable Development Goals (SDGs). In 2015, mental health was formally recognised as a core developmental priority (Votruba et al. 2016), although SDG 16 highlighted the importance of crime prevention and justice reform. Mental health and criminal behaviour are thus treated almost as parallel policy concerns. However, the extent and direction of their interrelationship remain unclear (Ozkan et al. 2019). Pathways between depression and anxiety on the one hand and criminal behaviour on the other are not yet well documented.

Depression and anxiety are among the most prevalent mental disorders worldwide and affect approximately one in eight people globally (Institute of Health Metrics and Evaluation 2022). Both are associated with socioeconomic disadvantage, social exclusion and criminal justice system involvement (Arias et al. 2022), but these are also disorders that may follow from each or all of these experiences. Societal costs of persistent offending are substantial, with the lifetime cost of a career offender estimated at up to $36 million (M. A. Cohen 1998; M. A. Cohen and Piquero 2009), so clarifying the direction and nature of associations between treatable mental health problems and crime is important for informing effective prevention and intervention strategies.

Previous studies suggest that internalising symptoms such as depression or anxiety may contribute to offending through impaired emotional regulation, impulsivity or maladaptive coping (Akse et al. 2007; Anderson et al. 2015). Others argue that criminal justice contact exacerbates psychological distress due to institutionalisation, stigma or life disruption (Marcotte and Markowitz 2011; Siennick 2007). It is also possible, however, that such associations—in either direction—reflect shared early‐life vulnerabilities, such as childhood adversity, family disruption or economic marginalisation, rather than being indicative of direct cause and effect (Moffitt 1993; Silver and Teasdale 2005; Rowe et al. 2010; Wiesner and Kim 2006; Kim et al. 2023; Kim et al. 2024). Indeed, both mental disorder and offending may stem from common developmental pathways rooted in early disadvantage (Moffitt 1993).

A key challenge in this literature is establishing causality. Many existing studies are cross‐sectional and cannot determine temporal ordering (Cook and Campbell 1979). Even longitudinal studies often fail to adequately adjust for confounders. Although randomised controlled trials (RCTs) may provide the strongest evidence for causal relationships, they are generally unfeasible or unethical in this context (Farrington and Welsh 2005). In such cases, propensity score matching (PSM) provides a quasiexperimental approach for approximating experimental control. By matching individuals on early‐life characteristics, such as adverse childhood experiences (ACEs), PSM allows for more rigorous comparison of exposed and nonexposed groups (Apel and Sweeten 2010; Farrington et al. 2020). Although it cannot eliminate unobserved confounding, PSM enhances the credibility of causal inference in observational data by reducing bias due to observed confounding, while acknowledging that unobserved heterogeneity cannot be eliminated.

In this study, we apply propensity score matching to data from the Cambridge Study in Delinquent Development (CSDD), a prospective cohort study that has followed 411 males from inner‐city London since childhood. It builds on prior work by Craig et al. (2018), who used PSM to assess the likelihood of arrest influencing later mental health. We extend this by testing bidirectional associations between mental disorder symptoms and crime and by examining depression and anxiety as distinct constructs. In doing so, we focus on two waves of data collection—at age 32 years and age 48 years. Our specific research questions were as follows:

1. Are depression and anxiety reported at age 32 associated with criminal convictions at age 48?
2. Are criminal convictions at age 32 associated with symptoms of depression or anxiety at age 48?

By adjusting for a wide range of childhood and adolescent risk factors, this study tests whether previously reported associations persist after accounting for shared vulnerabilities. In doing so, it contributes new evidence to a longstanding debate and provides insights for theory, policy and early intervention design.

## 2 Methods

### 2.1 Ethics

The Cambridge Study in Delinquent Development (CSDD) has received ethical approval for each wave of data collection from the Institute of Psychiatry, University of London, and the Institute of Criminology, University of Cambridge (Skinner and Farrington 2021, 213). No additional approvals were required for this secondary analysis of the data. The CSDD datasets offer a wealth of measures of childhood risk factors, which have already been tested in prior propensity score matching analyses (Craig et al. 2018).

### 2.2 Data and Sample

This study uses data from the Cambridge Study in Delinquent Development (CSDD), a prospective longitudinal study that has followed 411 boys in inner‐city London into and through adulthood. Participants were first assessed in 1961–1962 at age 8–9. On the basis of their fathers' occupations when they were aged 8, 94% of them could be described as ‘working‐class’ (Categories III, IV or V on the Registrar General's scale, describing skilled, semi‐skilled or unskilled workers), in comparison with the national figure of 78% at that time (Farrington et al. 2021, 280). The CSDD is one of the most detailed life‐course criminology datasets, with follow‐up interviews and data collection conducted periodically into participants' late adulthood. Participants have been interviewed nine times, at ages 8, 10, 14, 16, 18, 21, 25, 32 and 48. At all ages except 21 and 25, the aim was to interview all of those who were still alive. Because of inadequate funding, only about half of them were interviewed at age 21 and about a quarter at age 25. In all other interviews, however, a high proportion of those still living were interviewed: 405 (99%) at age 14, 399 (97%) at age 16, 389 (95%) at age 18, 378 (92%) at age 32 and 365 at age 48 (Farrington et al. 2021, 281). The detailed information in each wave and the high percentage of participants who remained in the study provided a rare opportunity to examine mental health and criminal behaviour across decades (Farrington et al. 2013).

This analysis focuses on associations between symptoms of depression and/or anxiety and criminal behaviour between ages 32 and 48. Childhood risk factors, measured prospectively from ages 8 to 18, were used to adjust for potential confounding factors, using propensity score matching.

### 2.3 Measures

*Symptoms of mental disorder* were assessed using the General Health Questionnaire (GHQ‐30), a validated self‐report screening tool covering nonpsychotic symptoms of psychiatric disorder in community samples (Goldberg 1972). It has 30 items, each rated on a four‐point Likert scale (0 = ‘not at all’ to 3 = ‘much more than usual’), with higher scores indicating greater distress and reflecting the state over a few weeks prior to the rating. For this study, GHQ‐30 data at ages 32 and 48 were analysed. Exploratory and confirmatory factor analyses confirmed reliable subscales for anxiety and depression (see analysis, below). It is important to note that the GHQ‐30 is not a diagnostic tool and principally serves here as an indicator of symptom severity and intrusion into recent daily life rather than necessarily a clinical disorder.

*Criminal behaviour* was assessed using official conviction records from age 10 to 48, accessed from the Central Record Office (CRO), the National Identification Service (NIS) and, from 1994, the Police National Computer (PNC). Only serious offences (e.g., theft, burglary and assault) were included; minor infractions (e.g., traffic infractions and drunkenness) were excluded to maintain focus on persistent and serious offending (Farrington et al. 2013). The most common offences included were thefts, burglaries and unauthorised taking of vehicles, although violence, vandalism, fraud and drug abuse were recorded too (Farrington et al. 2013, 13).

*Baseline childhood and adolescent risk factors for adult mental disorder or criminality* were measured using over 130 binary indicators from age 8 to 18, which covered individual characteristics such as impulsivity or educational attainment; family context, such as parental criminality or poverty; peer influence (e.g., delinquent friends); and self‐reported delinquency. Teacher and peer ratings provided additional perspectives on participants' behaviours (Farrington 2003; Farrington et al. 2015). These baseline risk factors were used to balance groups on background characteristics prior to further analyses.

### 2.4 Data Analytical Strategy

The analysis proceeded in four main stages:

First, we allowed for missing data. Given the longitudinal nature of the CSDD and the multiwave design of this study, some missing data were expected due to item nonresponse and participant attrition over time. Propensity score matching, the statistical tool applied in this study, cannot handle missing values (Thoemmes 2012). In fact, the proportion of missing data overall ranged from 0 to 0.27, and for relevant GHQ items measured at age 32, it was only around 0.08; for conviction variables, it was 0.01–0.10. Relevant GHQ‐30 items measured at age 48 had a slightly higher proportion of missing values, ranging between 0.16 and 0.17 (Schafer and Graham 2002). The proportion of missing data on background risk factors ranged from 0 to 0.27. The median proportion of missingness was 0.06.

Nevertheless, we managed missing values by imputation, using the expectation–maximisation (EM) algorithm (Dempster et al. 1977), which iteratively estimates missing values based on observed data patterns. This method generates estimates under the assumption that data are missing at random (Schafer and Graham 2002; Enders 2001; Allison 2002). Therefore, participants who had died or were permanently lost (e.g., through immigration to another country) to follow‐up by age 48 (*n* = 19) were excluded, as their data could not be reliably imputed.

Secondly, factor analysis (EFA) was conducted using data collected at ages 32 and 48 (Klainin‐Yobas and He 2014; Huppert et al. 1989; Chan 1985) to explore the underlying factor structure, followed by confirmatory factor analysis (CFA) to assess model fit, consistent with Gerbing and Anderson's (1988) recommended two‐step approach. Analyses were conducted using principal axis factoring (PAF) and principal component analysis (PCA), applying both orthogonal (varimax) and oblique (oblimin) rotations to identify the most stable and interpretable factor solutions (Costello and Osborne 2005). Factors were retained based on eigenvalues greater than 1 and factor loadings exceeding 0.30, following Kaiser's criterion (Kaiser 1960). Items with high cross‐loadings were examined to ensure conceptual clarity and internal consistency.

Thirdly, propensity score matching (PSM) was used to examine whether symptoms of depression or anxiety at age 32 were associated with criminal convictions at age 48. Although items of the GHQ‐30 are scored using conventional 0‐1‐2‐3 Likert scores, as done in the CSDD and adopted in this study, alternative binary scoring methods have also been used, including the ‘GHQ score’ (Goldberg and Hillier 1979), which rates the presence or absence of each problem (0‐0‐1‐1), ignoring differences in severity. Further, there is a third method known as the ‘corrected GHQ score’ (Goodchild and Duncan‐Jones 1985), which is based on the ‘GHQ score’, but treats only the ‘not at all’ response as an indication of bad health when it comes to negative items. The different scoring methods have been compared in several studies, but no clear conclusion has been reached as to which method provides the best sensitivity and specificity to detect ‘caseness’.

Regarding the purpose and aim of the study at hand, none of the scoring methods was particularly preferable because there are no standardised cut‐off points available for subscales of the GHQ‐30. Ultimately, all analyses of this study were based on the four‐point Likert scale because it was argued that both binary scoring methods would inevitably involve the loss of important information. It was decided to use all information provided by the items and only dichotomise the final total score to determine, more reliably, a cut‐off score for ‘caseness’. A binary classification is required for propensity score matching. To create this classification, participants were categorised as ‘positive for symptoms’ if their scores met or exceeded the 95th percentile (approximately 1.65 standard deviations above the mean). The application of the 95% confidence interval (CI) of the mean for choosing a cut‐off point is a commonly used method (Singh 2006) that identifies only the highest 5% of scores as positive cases. This threshold provides a conservative proxy for elevated symptom burden while acknowledging that the GHQ‐30 is a screening tool rather than a diagnostic instrument. These binary categories were used solely for analytic purposes in matching procedures and are not intended to denote clinical diagnoses.

In the fourth and final stage, propensity score matching was used to test for any relationship between having any criminal convictions at age 32 (yes/no) and depressive or anxiety symptoms at age 48.

Mean differences in outcomes between the ‘treated’ and ‘nontreated’ groups were assessed using the Mann–Whitney test for nonparametric samples.

*Propensity score matching* was used to reduce selection bias (due to careful matching of individuals on childhood risk factors) and strengthen the investigation of any possible causal inference from observational data (Rosenbaum and Rubin 1983). The propensity score represents the probability of being in the state—that is, either having symptoms of depression and/or anxiety or having criminal convictions, conditional on observed covariates. For example, when matching age 32 depressed versus nondepressed individuals (PSM Model 1; Supporting Information S1: Online Table S2) and age 32 anxious versus nonanxious individuals (PSM Model 2; Supporting Information S1: Online Table S3), we used individual‐level childhood risk factors (e.g., antiestablishment attitude and dishonesty), family‐level childhood risk factors (e.g., parental criminality and harsh discipline), school‐level factors (e.g., truancy and teacher‐rated aggressiveness) and behavioural risk factors (e.g., sexual promiscuity and antisocial behaviour). Childhood risk factors were measured across ages 8 to 18. After matching (in Models 1 and 2), we looked at differences in offending at age 48 between depressed/nondepressed and anxious/nonanxious groups accordingly, in line with Research Question 1. A similar analytical approach was followed for Research Question 2. Across the various PSM models used in this study, childhood risk factors were carefully selected based on earlier CSDD analyses on links between mental health and crime (Farrington 2003; Reising, Farrington, et al. 2019).

Propensity scores were estimated using logistic regression, and individuals were matched using nearest‐neighbour matching with a calliper of 0.20 standard deviations of the logit of the propensity score (Austin 2011; Ho et al. 2007). Covariate balance was evaluated using standardised mean differences (SMDs) with values < 0.25 considered acceptable balance (Rubin 2001). Unmatched individuals were excluded from the final analyses. Full prematching and postmatching covariate diagnostics are provided in Supporting Information S1: Online Tables.

Analyses were performed in SPSS IBM version 21 and R using the ‘psmatching’ plugin that was developed in SPSS/R by Thoemmes (2012).

## 3 Results

### 3.1 General Description of the Sample

In this study, we drew on the first five waves of the CSDD, which cover information regarding risk factors across ages eight to eighteen, as well as information on mental health and offending from follow‐ups in Wave 8 (at age 32) and Wave 9 (at age 48). Approximately 378 participants completed the age 32 follow‐up questionnaire (92% of baseline respondents), and 365 males completed the age 48 questionnaire (89% of baseline respondents).

Ten questions were used to measure anxiety. On these measures, in both Waves 8 and 9, the most frequently reported items were feeling constantly under strain (23% and 19%), having restless nights (both times 21%), losing sleep over worry (15% and 16%) and taking things hard (14% and 11%). Each of the four items used to measure depression was rated by fewer of the men, including feelings of worthlessness as a person (4% and 6%), experiencing life as entirely hopeless (4% and 5%), having bad nerves (2% and 6%) and thinking about life as not worth living (2% and 6%; see Supporting Information S1: Online Table S1 for more detail on both anxiety and depression scores).

### 3.2 Factor Structure of the GHQ‐30

Factor analyses supported the use of the GHQ‐30 to assess internalising symptoms at both age 32 and age 48. Sampling adequacy was high (Kaiser–Meyer–Olkin (KMO) values were 0.93 at age 32; 0.91 at age 48), exceeding the recommended threshold of 0.80 (Hutcheson and Sofroniou 1999).

PAF with direct oblimin rotation revealed a consistent four‐factor structure across both time points: anxiety, inadequate coping, depression and social dysfunction. These factors accounted for 45% of total variance at age 32 (see Table 1) and 49% at age 48 (see Table 2). Items loaded cleanly onto expected dimensions, with high communalities and conceptually coherent groupings. Internal consistency for the key subscales was high. Cronbach's alpha values for anxiety were 0.92 (age 32) and 0.94 (age 48), and for depression, they were 0.86 (age 32) and 0.87 (age 48). These statistics confirm that the GHQ‐30 reliably captures internalising symptom domains across time in this sample. These validated subscales were subsequently used to derive composite measures of depression and anxiety for the propensity score matching analyses.

**TABLE 1:** Four factor solution for the GHQ‐30—*age 32*.

Items		Factor loadings				Communality	Item–total corr.^a^
		Factor λ_1_	Factor λ_2_	Factor λ_3_	Factor λ_4_	h^2^	Rit
GHQ 14	Constantly under strain	**0.84**	0.07	−0.05	−0.04	0.65	0.74
GHQ 2	Lose sleep over worry	**0.74**	0.10	−0.01	−0.03	0.51	0.66
GHQ 21	Everything is getting on top	**0.74**	−0.09	0.12	0.08	0.67	0.79
GHQ 3	Restless nights	**0.68**	0.02	−0.05	−0.05	0.44	0.61
GHQ 18	Taking things hard	**0.63**	0.10	0.05	−0.10	0.43	0.61
GHQ 16	Life is a struggle	**0.62**	−0.10	0.08	0.03	0.49	0.66
GHQ 28	Feeling nervous	**0.59**	−0.05	0.29	0.03	0.61	0.73
GHQ 22	Feeling unhappy	**0.57**	−0.26	0.18	0.06	0.62	0.73
GHQ 19	Scared for no reason	**0.53**	−0.02	0.31	0.07	0.51	0.66
GHQ 15	Not overcome difficultly	**0.52**	−0.16	0.11	−0.03	0.45	0.63
GHQ 23	Losing confidence	**0.41**	−0.17	**0.38**	0.04	0.56	0.66
GHQ 7	Doing things well	−0.07	**0.66**	−0.05	0.03	0.52	0.63
GHQ 12	Playing a useful part	0.11	**0.63**	−0.10	0.15	0.51	0.59
GHQ 4	Able to keep busy	0.10	**0.61**	−0.03	−0.04	0.32	0.46
GHQ 13	Capable of decisions	0.08	**0.46**	−0.14	0.20	0.37	0.52
GHQ 8	Satisfied with task	−0.17	**0.45**	0.11	0.21	0.39	0.50
GHQ 1	Able to concentrate	**−0.28**	**0.29**	0.09	0.10	0.24	0.38
GHQ 26	Hopeful about the future	−0.11	**0.29**	−0.17	0.18	0.30	0.44
GHQ 6	Managing well	−0.07	**0.29**	0.06	−0.03	0.08	0.24
GHQ 5	Getting out	0.03	**0.22**	−0.13	0.14	0.13	0.29
GHQ 29	Life is not worth living	−0.03	0.09	**0.84**	−0.15	0.71	0.74
GHQ 24	You are worthless	0.12	−0.17	**0.74**	0.15	0.69	0.71
GHQ 25	Life is hopeless	0.18	−0.03	**0.65**	−0.06	0.62	0.73
GHQ 30	Nerves are too bad	0.20	0.06	**0.57**	−0.06	0.48	0.62
GHQ 10	Easy to get on with others	−0.06	−0.06	−0.08	**0.73**	0.56	0.60
GHQ 11	Chatting with others	0.06	0.02	0.05	**0.49**	0.23	0.33
GHQ 9	Feel warmth/affection	−0.04	0.03	−0.05	**0.45**	0.25	0.45
GHQ 27	Feeling happy	−0.14	0.20	−0.17	**0.38**	0.44	0.52
GHQ 17	Enjoy daily activities	−0.32	0.19	0.10	**0.35**	0.38	0.46
GHQ 20	Able to face problems	−0.04	**0.24**	**−0.24**	**0.24**	0.32	0.41
Eigenvalues		7.58	4.95	5.71	3.81	22.05	—
% of variance		31.40	7.22	4.05	2.35	—	—
Alpha		0.92	0.77	0.86	0.72	—	—

*Note:* Basis: 392 people, total variance explained: 45.01%. Extraction method: principal axis factoring, coefficients after ‘direct oblimin’ rotation. Bold values indicate the highest factor loading for each item, representing the factor on which the item loads most strongly and aiding interpretation of the factor structure.

^a^ The item–total correlation coefficients stem from individual reliability analyses of the four factors.

**TABLE 2:** Four factor solution for the GHQ‐30—*age 48*.

Items		Factor loadings				Communality	Item–total corr.^a^
		Factor λ_1_	Factor λ_2_	Factor λ_3_	Factor λ_4_	h^2^	Rit
GHQ 3	Restless nights	**0.85**	0.03	0.14	−0.04	0.60	0.72
GHQ 2	Lose sleep over worry	**0.83**	−0.03	0.12	−0.05	0.59	0.72
GHQ 21	Everything is getting on top	**0.75**	0.07	−0.09	0.03	0.65	0.78
GHQ 22	Feeling unhappy	**0.75**	0.01	−0.14	0.01	0.70	0.81
GHQ 14	Constantly under strain	**0.74**	0.07	−0.01	0.11	0.54	0.71
GHQ 16	Life is a struggle	**0.68**	−0.04	−0.14	−0.14	0.66	0.78
GHQ 18	Taking things hard	**0.67**	−0.03	−0.07	0.01	0.51	0.69
GHQ 23	Losing confidence	**0.63**	0.04	**−0.34**	0.06	0.76	0.82
GHQ 28	Feeling nervous	**0.61**	0.11	−0.25	0.04	0.62	0.74
GHQ 19	Scared for no reason	**0.61**	−0.01	−0.23	−0.04	0.60	0.74
GHQ 15	Not overcome difficultly	**0.55**	−0.13	−0.14	−0.17	0.47	0.65
GHQ 10	Easy to get on with others	0.01	**0.73**	−0.09	0.10	0.49	0.60
GHQ 11	Chatting with others	−0.03	**0.65**	0.02	0.09	0.37	0.51
GHQ 12	Playing a useful part	−0.02	**0.59**	−0.09	−0.10	0.45	0.61
GHQ 6	Managing well	0.07	**0.57**	0.07	0.02	0.31	0.49
GHQ 8	Satisfied with task	0.02	**0.54**	−0.13	−0.17	0.47	0.62
GHQ 13	Capable of decisions	0.04	**0.50**	0.06	−0.26	0.44	0.59
GHQ 5	Getting out	−0.02	**0.45**	0.11	0.03	0.19	0.36
GHQ 9	Feel warmth/affection	−0.03	**0.44**	−0.04	−0.20	0.33	0.51
GHQ 17	Enjoy daily activities	0.16	**0.39**	−0.02	−0.17	0.29	0.46
GHQ 7	Doing things well	−0.04	**0.38**	−0.14	−0.33	0.44	0.56
GHQ 4	Able to keep busy	−0.11	**0.31**	−0.08	−0.26	0.26	0.45
GHQ 24	You are worthless	0.14	0.09	**−0.79**	0.12	0.36	0.76
GHQ 25	Life is hopeless	0.19	0.00	**−0.75**	−0.01	0.76	0.81
GHQ 29	Life is not worth living	0.08	−0.08	**−0.70**	−0.06	0.57	0.72
GHQ 30	Nerves are too bad	0.29	−0.10	**−0.49**	−0.08	0.51	0.64
GHQ 27	Feeling happy	0.12	0.00	0.12	**−0.76**	0.57	0.54
GHQ 26	Hopeful about the future	0.13	−0.01	−0.02	**−0.57**	0.77	0.43
GHQ 20	Able to face problems	−0.12	0.14	−0.08	**−0.43**	0.28	0.39
GHQ 1	Able to concentrate	−0.04	**0.13**	−0.07	**−0.18**	0.08	0.14
Eigenvalues		7.97	4.06	6.11	3.43	21.57	—
% of variance		29.29	14.19	2.92	2.38	—	—
Alpha		0.94	0.84	0.87	0.59	—	—

*Note:* Basis: 392 people. Total variance explained: 48.78%. Extraction method: principal axis factoring. Coefficients after ‘direct oblimin’ rotation. Bold values indicate the highest factor loading for each item, representing the factor on which the item loads most strongly and aiding interpretation of the factor structure.

^a^ The item–total correlation coefficients stem from individual reliability analyses of the four factors.

### 3.3 Depression and Anxiety Symptoms

Composite scores on the GHQ ranged from 0 to 12 for depression and 0 to 30 for anxiety, with higher scores reflecting greater symptom severity (see Supporting Information S1: Online Table S1). Based on composite scores, symptoms of internalising problem behaviours were highly correlated with each other. There was a significant association between showing depressive as well as anxious symptoms at age 32, χ^2^ (1) = 38.91, *p* < 0.001. The same was true for internalising problem behaviours at age 48 (χ^2^ (1) = 156.17, *p* < 0.001). Internalising symptoms also appeared to be stable over time. As one example, age 32 depression was highly correlated with age 48 depression (χ^2^ (1) = 26.98, *p* < 0.001).

For the purpose of propensity score matching, participants were classified as having elevated symptoms if their scores met or exceeded the 95th percentile for each subscale.

### 3.4 Depression at Age 32 and Convictions at Age 48

In the full sample prior to propensity score matching, men with elevated depressive symptoms at age 32 (i.e., those at or above the 95th percentile) had significantly more convictions at age 48 (*M* = 0.57) than those without elevated symptoms (*M* = 0.40; *U* = 4491.00, *z* = 2.25, *p* < 0.05, *r* = 0.11).

Using propensity score matching, 19 men with elevated depressive symptoms (i.e., at or above the 95th percentile) were matched to 19 without elevated depression according to childhood and adolescent risk factors for both offending and mental disorder. Covariate balance was strong, with only one variable (‘criminal parent’ at age 8) slightly exceeding the standard mean difference threshold (*d* = −0.323). Detailed prematching and postmatching statistics are reported in Supporting Information S1: Online Table S2.

The overall chi‐squared balance test was not significant, confirming overall group equivalence (χ^2^ (29) = 28.19, *p* = 0.508). Because, according to propensity matching, the two groups did not differ in conviction risk measures prior to becoming depressive, the impact of experiencing depression at age 32 on later offending behaviour at age 48 was examined using the Mann–Whitney test for nonparametric models. In this matched sample (*N* = 38), conviction rates no longer differed significantly between the depressed and nondepressed groups (*M* = 0.60 v *M* = 0.59; *U* = 201.00, *z* = 0.71, *p* = 0.563, *r* = 0.04), suggesting that the association observed in the *full unmatched* sample was likely to be due to shared early‐life risk factors rather than a direct effect of depression on later offending. Summary statistics for both the full and matched samples are presented in Table 3.

**TABLE 3:** Comparison of the mean conviction rates between depressive and nondepressive study males before and after matching.

	Min.	Max.	M	SD	*n*
Convictions between ages 33 and 48					
Full sample age 32					
Depressive group	0	4	**0.57**	0.57	20
Nondepressive group	0	12	**0.40**	1.34	372
*z* = 2.25, *p* < 0.05					
Matched sample age 32					
Depressive group	0	4	**0.60**	1.08	19
Nondepressive group	0	4	**0.59**	0.59	19
*z* = 0.71, *p* = 0.563					

*Note:* Bold values indicate the highest factor loading for each item, representing the factor on which the item loads most strongly and aiding interpretation of the factor structure.

### 3.5 Anxiety at Age 32 and Convictions at Age 48

In the full sample, before propensity matching, a crude group mean comparison between age 32 males with and without anxiety symptoms (i.e., those at or above the 95th percentile) revealed that experiencing anxiety at age 32 was positively associated with conviction rates at age 48. Those with elevated symptoms had higher subsequent conviction rates than those below the threshold; men with elevated anxiety symptoms at age 32 had significantly more convictions at age 48 (*M* = 0.80) than those without (*M* = 0.39; *U* = 4412.00, *z* = 2.022, *p* < 0.05, *r* = 0.10; see also Table 4).

**TABLE 4:** Comparison of the mean conviction rates between anxious and nonanxious study males before and after matching.

	Min.	Max.	M	SD	*n*
Convictions between ages 33 and 48					
Full sample age 32					
Anxious group	0	4	**0.80**	1.29	20
Nonanxious group	0	12	**0.39**	1.32	372
*z* = 2.02, *p* < 0.05					
Matched sample age 32					
Anxious group	0	4	**0.85**	1.32	19
Nonanxious group	0	8	**0.64**	1.89	19
*z* = 0.79, *p* = 0.506					

*Note:* Bold values indicate the highest factor loading for each item, representing the factor on which the item loads most strongly and aiding interpretation of the factor structure.

After propensity score matching, however, confirming similarity on early risk factors for offending (χ^2^ (32) = 25.07, *p* = 0.803; see Supporting Information S1: Online Table S3 for details), the relationship between anxiety at age 32 and new offending in the subsequent 16 years was tested using the Mann–Whitney test for nonparametric models in this group of 38 men. The anxious/nonanxious groups no longer differed in conviction rates between ages 32 and 48 (*M* = 0.85 for anxious vs. *M* = 0.64 for nonanxious; *U* = 204.00, *z* = 0.79, *p* = 0.506, *r* = 0.13). These findings again suggest that shared, earlier vulnerabilities better account for the later offending. However, the small sample size in the matched groups may have reduced statistical power.

### 3.6 Convictions at Age 32 and Depression at Age 48

In the third set of analyses, the relationship between having received at least one conviction up to age 32 and evidence of depression 16 years later was examined.

A preliminary group mean comparison of depression at age 48 between men who had received at least one conviction before age 32 and those who had not was conducted. There was no difference between the groups in later depression (pre‐age 32 convictions group mean depression score = 1.59; unconvicted men mean depression score = 1.20; *U* = 17,432.50, *z* = 1.43, *p* = 0.152, *r* = 0.07).

Although this initial analysis did not reveal any significant relationship, propensity score matching was also applied to check that the two matched groups did not differ in prior depression risk. Detailed prematching and postmatching statistics are reported in Supporting Information S1: Online Table S4. After establishing group equivalence in prior depression risk, the relationship between age 32 convictions and age 48 depression was examined using the Mann–Whitney test for nonparametric models. The average depression levels at age 48 in men with criminal records (*M* = 1.20) did not differ significantly from those in nonconvicted men (*M* = 2.06; *U* = 5572.00, *z* = −0.78, *p* = 0.438, *r* = −0.05); in fact, there was a slight trend towards the convicted men having lower levels of depression (see also Table 5).

**TABLE 5:** Comparison of the mean depression score between convicted and nonconvicted study males before and after matching.

	Min.	Max.	M	SD	*n*
Depression score at age 48					
Full sample age 32					
Convicted group	0	10	**1.59**	2.31	117
Nonconvicted group	0	12	**1.20**	2.08	275
*z* = 1.43, *p* = 0.152					
Matched sample age 32					
Convicted group	0	10	**1.64**	2.27	113
Nonconvicted group	0	12	**2.06**	3.02	101
*z* = −0.78, *p* = 0.438					

*Note:* Bold values indicate the highest factor loading for each item, representing the factor on which the item loads most strongly and aiding interpretation of the factor structure.

### 3.7 Convictions at Age 32 and Anxiety at Age 48

The exploration of temporal relationships between offending and later mental state problems was then extended to anxiety. In the full sample, a crude group mean comparison between men aged 32 who had had any criminal conviction by that age and those who had not showed little difference in the expression of anxiety at age 48 (conviction group *M* = 8.69; conviction‐free group *M* = 7.68; *U* = 17,644.50, *z* = 1.52, *p* = 0.129, *r* = 0.08).

Although this initial analysis did not reveal any significant association, propensity score matching was again applied to check for possible confounding predisposing factors. For a complete list of all risk factors included in this fourth PSM analysis, see Supporting Information S1: Online Table S5. After matching, a Mann–Whitney test confirmed that there was no relationship between having had a criminal conviction by age 32 and being anxious at age 48 (criminal record group *M* = 8.58; nonconvicted group *M* = 9.33; *U* = 5723.50, *z* = −0.44, *p* = 0.660, *r* = −0.03). As with depression, there was some indication that men with prior convictions were slightly less anxious at age 48. Summary statistics for both the full and matched samples are presented in Table 6.

**TABLE 6:** Comparison of the mean anxiety score between convicted and nonconvicted study males before and after matching.

	Min.	Max.	M	SD	*n*
Anxiety score at age 48					
Full sample age 32					
Convicted group	0	30	**8.69**	6.25	117
Nonconvicted group	0	28	**7.68**	5.74	275
*z* = 1.52, *p* = 0.129					
Matched sample age 32					
Convicted group	0	30	**8.58**	6.00	114
Nonconvicted group	0	27	**9.33**	7.06	104
*z* = −0.44, *p* = 0.660					

*Note:* Bold values indicate the highest factor loading for each item, representing the factor on which the item loads most strongly and aiding interpretation of the factor structure.

## 4 Discussion

Analyses of data on depression or anxiety, as measured by the General Health Questionnaire, and much later offending suggest a relationship between depression and/or anxiety at age 32 and new criminal offending by age 48, but this is mainly accounted for by risk factors for offending recorded in childhood. There was no relationship between offending up to age 32 and much later anxiety or depression at age 48, whether or not allowing for possible confounding by earlier risk factors. It is well recognised that adverse childhood experiences (ACEs) are linked with compromised health and social outcomes in adulthood (e.g., Belsky et al. 2020; Kaplow and Widom 2007), with some earlier research also pointing to a clear dose–response effect between childhood risk factors and compromised life success (Craig et al. 2017). Nevertheless, it might be expected that adult breaks with health and offending might have some additional interrelationships, but our findings suggest that this is unlikely to reflect a direct relationship for anxiety and depression.

Although prior research highlights bidirectional links between internalising symptoms and externalising problem behaviours (De Coster and Heimer 2001; Hagan and Foster 2003; Beyers and Loeber 2003), few studies have applied rigorous quasiexperimental methods to assess whether such associations may be largely accounted for by early‐life adversities. By using propensity score matching, our study adds to the literature by approximating experimental control in a naturalistic setting. Although PSM does not establish causality, it reduced selection bias in between‐individual analyses by balancing known confounders across groups and thereby strengthening confidence in the interpretation of results (Apel and Sweeten 2010; Farrington et al. 2020). The proposition that childhood delinquents and young adult offenders are more prone to develop health problems is not new (Gottfredson and Hirschi 1990, 94–96; Robins 1978), and associations between internalising and externalising symptoms are typically regarded as the result of confounding factors (Testa and Semenza 2020). In this paper, analyses are based on associations between criminality and mental health in later adulthood (ages 32–48). Studies examining the reciprocal effects between internalising and externalising symptoms are also available, although less common (Stogner et al. 2014), and our paper adds to this gap in scientific knowledge regarding reciprocal associations.

Despite significant long‐term associations between offending trajectories and mental health (Reising, Ttofi, et al. 2019), our findings suggest that any relationship between depression and anxiety at age 32 and subsequent offending may be best accounted for by early‐life experiences creating the conditions for both. Specifically, small significant differences observed across the *full unmatched* sample did not hold in *matched‐group* comparisons that incorporated early childhood developmental vulnerabilities. In addition, we found no link at all between offending at age 32 and subsequent depression/anxiety in either the full‐unmatched or the matched group comparisons. This does not mean that the more proximate relationships are unimportant, but rather that early intervention to minimise the impact of adverse childhood events is likely to have maximum preventive value for offending as well as mental health problems.

Effective prevention may require earlier intervention targeting the shared roots of both outcomes. Interventions to prevent childhood adversities and associated (health and other) impairments are available and show promise (J. A. Cohen et al. 2006; MacMillan et al. 2009), although they should be critically assessed within their relevant context (Farrington et al. 2017).

### 4.1 Limitations

The Cambridge Study is a major longitudinal study with prospective measures of ACEs, offending and mental health. The prospective nature of data collection is one of its major strengths (Coleman et al. 2024; Farrington 2003). For all its strengths as a particularly long‐lasting longitudinal prospective study, there are inevitable limitations in the data. First, the sample is comprised exclusively of White, working‐class males from inner London, which may limit generalisability to other populations. Secondly, criminal behaviour was assessed using official conviction data, which may underestimate true offending (Farrington et al. 2014). In fact, a whole prior issue of this journal—CBMH—was dedicated to longitudinal research on convergence and divergence in self‐reported and officially reported criminal behaviour (Farrington and Ttofi 2014). Thirdly, the GHQ‐30 measures recent symptoms of distress and does not provide measures of enduring or clinically diagnosed mental disorders. Although subscales were validated in this study through factor analysis and were consistent across age 32 and age 48 time points, the GHQ‐30 reflects short‐term symptom fluctuation and may not capture chronic or severe mental illness. Further, the use of a conservative statistical threshold (top 5%) to identify ‘caseness’ was a valid methodological necessity for propensity score matching (Singh 2006) but does not equate to clinical diagnosis. The sample size was rather small for detecting most major mental disorders; indeed, no man had schizophrenia, and, after propensity matching, numbers with the more common depression or anxiety were very small indeed. Finally, the measures were taken very far apart; few mental health professionals, if any at all, would expect even well‐substantiated clinical depression or anxiety as a clear disorder measured at one point in time to have an impact on offending up to 16 years later, or the converse. It would have been ideal if more frequent follow‐up assessments were available. Nevertheless, the finding that early antecedents common to features of interest may better explain apparent associations than any later, more direct relationship is an important one for immediate practice and for early prevention.

Future research should expand on this design by incorporating more frequent measurements, examining within‐individual change and exploring a broader range of symptom domains (Farrington et al. 2002; Hemphill et al. 2015). Including more diverse samples (Gaba et al. 2022) and mixed‐methods approaches could offer further insights into the pathways linking mental health and offending. Longitudinal studies with multiple waves may also help clarify whether fluctuations in mental disorders or their symptoms are temporally related to behavioural outcomes. Cross‐sector collaboration, across health, education and justice, will be vital for developing effective early interventions that address the causes of both mental health problems and crime (Gebo 2022; Shepherd and Farrington 1993) and ensure better access for vulnerable populations (Hardin et al. 2020).

## Supporting information


Supporting Information S1

## Data Availability

The data that support the findings of this study are available from the Cambridge Study in Delinquent Development (CSDD). Access to these data is subject to approval by the study directors and relevant data access procedures.
