# Supplementary material for: Depression, Anxiety and Criminal Behaviour Between Ages 32 and 48: A Propensity Score Matching Analysis From the Cambridge Study in Delinquent Development
Source: Crim Behav Ment Health. 2026 May 6;36(5):236–46. doi: 10.1002/cbm.70036 (PMC13664659; doi:10.1002/cbm.70036)
Supplement: Supplementary file 1 [file CBM-36-236-s001.docx]

**Online Materials for paper “Depression, Anxiety and Criminal Behaviour Between Ages 32 and 48: A Propensity Score Matching Analysis from the Cambridge Study in Delinquent Development” by Kim Reising and Maria M. Ttofi**

***Online Table 1:* Item summary statistics of the anxiety and depression scales**

|  | | | |  |  | | | **Age 32** | | **Age 48** | | |  |
| --- | --- | --- | --- | --- | --- | --- | --- | --- | --- | --- | --- | --- | --- |
|  | | | | | | **min** | **max** | **M** | **SD** | **M** | **SD** | **n** | |
| **Depression** | | | | | | **0** | **12** | **0.86** | **1.72** | **1.32** | **2.15** | **392** | |
|  | GHQ 24 | worthless person | | | | 0 | 3 | 0.28 | 0.56 | 0.36 | 0.65 | 392 | |
|  | GHQ 25 | life entirely hopeless | | | | 0 | 3 | 0.24 | 0.54 | 0.34 | 0.63 | 392 | |
|  | GHQ 30 | nerves are too bad | | | | 0 | 3 | 0.19 | 0.49 | 0.32 | 0.63 | 392 | |
|  | GHQ 29 | life not worth living | | | | 0 | 3 | 0.15 | 0.47 | 0.30 | 0.62 | 392 | |
| **Anxiety** | | | | | | **0** | **30** | **7.46** | **5.51** | **7.99** | **5.91** | **392** | |
|  | GHQ 14 | constantly under strain | | | | 0 | 3 | 1.00 | 0.76 | 1.00 | 0.76 | 392 | |
|  | GHQ 18 | taking things hard | | | | 0 | 3 | 0.87 | 0.70 | 0.88 | 0.69 | 392 | |
|  | GHQ 3 | restless nights | | | | 0 | 3 | 0.87 | 0.84 | 0.97 | 0.85 | 392 | |
|  | GHQ 2 | lose sleep over worry | | | | 0 | 3 | 0.80 | 0.76 | 0.89 | 0.81 | 392 | |
|  | GHQ 16 | life a struggle | | | | 0 | 3 | 0.78 | 0.69 | 0.78 | 0.69 | 392 | |
|  | GHQ 21 | everything getting on top | | | | 0 | 3 | 0.74 | 0.74 | 0.82 | 0.78 | 392 | |
|  | GHQ 15 | Not overcome difficulties | | | | 0 | 3 | 0.72 | 0.72 | 0.72 | 0.72 | 392 | |
|  | GHQ 22 | feeling unhappy | | | | 0 | 3 | 0.70 | 0.81 | 0.78 | 0.81 | 392 | |
|  | GHQ 28 | feeling nervous | | | | 0 | 3 | 0.50 | 0.68 | 0.52 | 0.66 | 392 | |
|  | GHQ 19 | scared for no reason | | | | 0 | 3 | 0.49 | 0.67 | 0.54 | 0.71 | 392 | |
|  |  | |  | | |  |  |  |  |  |  |  | |

***Online Table 2:* List of covariates included in the first PSM analysis and detailed balance information for the Age 32 depressive and non-depressive group before and after matching**

| **Risk factors and  time of measurement (age)** | **Full (unmatched) Sample** | | | **PSM I** |
| --- | --- | --- | --- | --- |
|  | depressive  (n = 20) | non-depressive  (n = 372) | Standard  difference² | Standard. difference² |

*Individual*

| 8 | troublesome | 0.35 | 0.20 | 0.30 | **-0.22** |
| --- | --- | --- | --- | --- | --- |
| 10 | dishonest | 0.40 | 0.25 | 0.31 | **0.21** |
| 10 | vulnerable | 0.30 | 0.15 | 0.32 | **-0.22** |
| 10 | unpopular | 0.45 | 0.32 | 0.27 | **0.10** |
| 10 | lacks concentration / restless | 0.25 | 0.19 | 0.13 | **-0.12** |
| 18 | anti-police attitude | 0.15 | 0.23 | -0.22 | **-0.14** |
| 18 | antiestablishment attitude | 0.35 | 0.25 | 0.20 | **0.00** |

*Family*

| 8 | Parental criminality | 0.35 | 0.26 | 0.19 | **-0.32** |
| --- | --- | --- | --- | --- | --- |
| 10 | parents separated | 0.30 | 0.22 | 0.18 | **0.00** |
| 10 | broken home | 0.95 | 0.88 | 0.31 | **-0.24** |
| 10 | harsh discipline | 0.25 | 0.28 | -0.07 | **0.12** |
| 10 | father involvement | 0.50 | 0.28 | 0.44 | **-0.10** |
| 10 | poor supervision | 0.20 | 0.19 | 0.04 | **0.13** |
| 18 | poor relation with parents | 0.45 | 0.20 | 0.48 | **0.21** |

*School*

| 10 | truancy | 0.10 | 0.06 | 0.13 | **0.17** |
| --- | --- | --- | --- | --- | --- |
| 14 | delinquent friends | 0.35 | 0.24 | 0.23 | **0.00** |
| 14 | teacher and self-reported truancy | 0.20 | 0.12 | 0.19 | **-0.13** |
| 14 | teacher rated aggressive | 0.45 | 0.32 | 0.27 | **0.10** |
| 14 | teacher rated lacks concentration | 0.40 | 0.25 | 0.29 | **0.00** |
| 18 | no exams taken | 0.45 | 0.32 | 0.27 | **0.10** |
| 18 | unemployed | 0.40 | 0.25 | 0.29 | **0.00** |

*Behavioural*

| 14 | antisocial combined | 0.09 | 0.21 | 0.09 | **0.00** |
| --- | --- | --- | --- | --- | --- |
| 18 | antisocial combined | 0.20 | 0.24 | -0.09 | **0.00** |
| 18 | binge drinker | 0.25 | 0.20 | 0.11 | **-0.24** |
| 18 | promiscuous sex | 0.30 | 0.29 | 0.03 | **0.13** |
| 18 | irresponsible sex | 0.25 | 0.21 | 0.12 | **0.24** |
| 18 | drunk driver | 0.25 | 0.21 | 0.09 | **0.12** |
| 18 | drug user | 0.40 | 0.30 | 0.20 | **0.00** |
| 18 | convictions | 2.85 | 1.68 | 0.25 | **0.16** |

Balance checks: all absolute standardised differences in means should be less than 0.25 for each variable (see Rubin 2001)

***Online Table 3:* List of covariates included in the second PSM analysis and detailed balance information for the Age 32 anxious and non-anxious group before and after matching**

| **Risk factors and  time of measurement (age)** | **Full (unmatched) Sample** | | | **PSM II** |
| --- | --- | --- | --- | --- |
|  | anxious  (n = 20) | non- anxious  (n = 372) | Standard  difference² | Standard. difference² |

*Individual*

| 10 | dishonest | 0.30 | 0.25 | 0.11 | **0.00** |
| --- | --- | --- | --- | --- | --- |
| 10 | vulnerable | 0.30 | 0.15 | 0.32 | **0.11** |
| 10 | unpopular | 0.30 | 0.32 | -0.05 | **-0.11** |
| 10 | lacks concentration / restless | 0.25 | 0.19 | 0.13 | **0.00** |
| 10 | difficult to discipline | 0.20 | 0.21 | -0.03 | **0.13** |
| 18 | anti-police attitude | 0.40 | 0.22 | 0.36 | **0.00** |
| 18 | antiestablishment attitude | 0.45 | 0.24 | 0.40 | **0.00** |

*Family*

| 8 | Parental criminality | 0.45 | 0.25 | 0.39 | **0.21** |
| --- | --- | --- | --- | --- | --- |
| 10 | parents separated | 0.25 | 0.22 | 0.07 | **-0.12** |
| 10 | broken home | 0.10 | 0.12 | -0.05 | **-0.17** |
| 10 | harsh discipline | 0.40 | 0.27 | 0.26 | **-0.21** |
| 10 | Parental disharmony | 0.50 | 0.28 | 0.42 | **0.00** |
| 18 | poor relation with parents | 0.40 | 0.21 | 0.38 | **0.00** |

*School*

| 10 | truancy | 0.20 | 0.05 | 0.36 | **0.00** |
| --- | --- | --- | --- | --- | --- |
| 14 | delinquent friends | 0.45 | 0.77 | -0.63 | **0.10** |
| 14 | teacher and self-reported truancy | 0.50 | 0.27 | 0.45 | **0.00** |
| 14 | teacher rated aggressive | 0.45 | 0.31 | 0.27 | **0.21** |
| 14 | teacher rated lacks concentration | 0.45 | 0.25 | 0.39 | **0.00** |
| 14 | teacher rated frequent truant | 0.35 | 0.17 | 0.37 | **-0.11** |
| 14 | teacher rated daring | 0.10 | 0.13 | -0.09 | **0.00** |
| 18 | unemployed | 0.20 | 0.21 | -0.03 | **-0.13** |
| 18 | stayed at school | 0.25 | 0.40 | -0.33 | **0.00** |

*Behavioural*

| 14 | antisocial combined | 0.30 | 0.22 | 0.17 | **-0.11** |
| --- | --- | --- | --- | --- | --- |
| 18 | antisocial combined | 0.35 | 0.23 | 0.25 | **0.00** |
| 18 | binge drinker | 0.35 | 0.20 | 0.31 | **-0.11** |
| 18 | promiscuous sex | 0.45 | 0.28 | 0.34 | **0.10** |
| 18 | irresponsible sex | 0.20 | 0.20 | 0.00 | **0.13** |
| 18 | drunk driver | 0.30 | 0.21 | 0.19 | **-0.11** |
| 18 | drug user | 0.50 | 0.30 | 0.40 | **-0.10** |

Balance checks: all absolute standardised differences in means should be less than 0.25 for each variable (see Rubin 2001).

***Online Table 4:* List of covariates included in the third PSM analysis and detailed balance information for the Age 32 convicted and non-convicted group before and after matching**

| **Risk factors and  time of measurement (age)** | **Full (unmatched) Sample** | | | **PSM III** |
| --- | --- | --- | --- | --- |
|  | convicted  (n = 113) | non- convicted  (n = 279) | Standard  difference² | Standard. difference² |

*Individual*

| 8 | troublesome | 0.32 | 0.17 | 0.32 | **0.04** |
| --- | --- | --- | --- | --- | --- |
| 10 | vulnerable | 0.31 | 0.09 | 0.47 | **-0.02** |
| 10 | small | 0.21 | 0.16 | 0.12 | **0.09** |
| 14 | neuroticism | 0.32 | 0.21 | 0.23 | **0.04** |
| 18 | neuroticism | 0.29 | 0.24 | 0.11 | **0.02** |

*Family*

| 8 | Parental criminality | 0.42 | 0.19 | 0.47 | **0.09** |
| --- | --- | --- | --- | --- | --- |
| 8 | behaviour problems sibling | 0.49 | 0.32 | 0.33 | **-0.06** |
| 8 | large family size | 0.38 | 0.18 | 0.41 | **-0.06** |
| 8 | low family income | 0.34 | 0.19 | 0.32 | **-0.14** |
| 8 | marital disharmony | 0.30 | 0.19 | 0.23 | **-0.04** |
| 8 | physical neglect | 0.23 | 0.08 | 0.36 | **-0.18** |
| 10 | parents separated | 0.32 | 0.18 | 0.29 | **0.10** |
| 10 | harsh discipline | 0.37 | 0.24 | 0.27 | **0.12** |
| 10 | father involvement | 0.37 | 0.25 | 0.24 | **-0.08** |
| 10 | poor supervision | 0.29 | 0.14 | 0.33 | **0.18** |
| 10 | Parental disharmony | 0.61 | 0.75 | -0.28 | **0.02** |
| 10 | poor housing | 0.48 | 0.33 | 0.30 | **-0.07** |
| 10 | father unemployed | 0.19 | 0.10 | 0.22 | **-0.07** |
| 10 | nervous mother | 0.36 | 0.31 | 0.11 | **-0.06** |
| 10 | low SES | 0.27 | 0.16 | 0.23 | **0.02** |
| 14 | low SES | 0.35 | 0.32 | 0.08 | **0.00** |
| 14 | large family size | 0.33 | 0.17 | 0.33 | **0.02** |
| 18 | poor relation with parents | 0.30 | 0.18 | 0.26 | **-0.04** |

*School*

| 8 | low non-verbal IQ | 0.35 | 0.22 | 0.29 | **-0.06** |
| --- | --- | --- | --- | --- | --- |
| 10 | truancy | 0.12 | 0.04 | 0.24 | **-0.20** |
| 10 | low attainment | 0.42 | 0.17 | 0.50 | **-0.04** |
| 10 | delinquent school | 0.27 | 0.18 | 0.19 | **-0.02** |
| 14 | delinquent friends | 0.42 | 0.17 | 0.51 | **-0.04** |
| 14 | teacher rated frequent truant | 0.34 | 0.11 | 0.47 | **0.00** |
| 14 | low verbal IQ | 0.35 | 0.18 | 0.36 | **-0.14** |
| 14 | teacher rated anxious | 0.13 | 0.08 | 0.17 | **-0.11** |
| 14 | teacher and self-reported anxiety | 0.31 | 0.28 | 0.06 | **0.00** |
| 14 | teacher rated unpopular | 0.20 | 0.15 | 0.13 | **0.00** |
| 18 | no exams taken | 0.69 | 0.44 | 0.54 | **0.14** |
| 18 | unemployed | 0.35 | 0.16 | 0.39 | **0.02** |
| 18 | unskilled manual job | 0.30 | 0.10 | 0.43 | **0.10** |
| 18 | unstable job record | 0.38 | 0.19 | 0.40 | **0.02** |
| 18 | reading ability | 0.12 | 0.05 | 0.23 | **-0.11** |

Balance checks: all absolute standardised differences in means should be less than 0.25 for each variable (see Rubin 2001)

***Online Table 5:* List of covariates included in the fourth PSM analysis and detailed balance information for the Age convicted and non-convicted group before and after matching**

| **Risk factors and  time of measurement (age)** | **Full (unmatched) Sample** | | | **PSM IV** |
| --- | --- | --- | --- | --- |
|  | convicted  (n = 113) | non-convicted  (n = 279) | Standard  difference² | Standard. difference² |

*Individual*

| 10 | vulnerable | 0.31 | 0.09 | 0.47 | **0.02** |
| --- | --- | --- | --- | --- | --- |
| 10 | neuroticism | 0.31 | 0.30 | 0.01 | **0.00** |
| 14 | neuroticism | 0.32 | 0.21 | 0.23 | **0.10** |

*Family*

| 8 | Parental criminality | 0.42 | 0.19 | 0.47 | **-0.04** |
| --- | --- | --- | --- | --- | --- |
| 8 | behaviour problems sibling | 0.49 | 0.32 | 0.33 | **0.04** |
| 10 | parents separated | 0.32 | 0.18 | 0.29 | **-0.12** |
| 10 | father involvement | 0.37 | 0.25 | 0.24 | **0.04** |
| 10 | poor supervision | 0.29 | 0.14 | 0.33 | **-0.06** |
| 10 | Parental disharmony | 0.61 | 0.75 | -0.28 | **-0.04** |
| 10 | poor housing | 0.48 | 0.33 | 0.30 | **-0.07** |

*School*

| 8 | low non-verbal IQ | 0.35 | 0.22 | 0.29 | **0.02** |
| --- | --- | --- | --- | --- | --- |
| 8 | low interest in education | 0.26 | 0.12 | 0.31 | **0.04** |
| 10 | truancy | 0.12 | 0.04 | 0.24 | **-0.03** |
| 10 | low attainment | 0.42 | 0.17 | 0.50 | **0.06** |
| 14 | teacher and self-reported truancy | 0.50 | 0.20 | 0.59 | **0.02** |
| 14 | teacher rated frequent truant | 0.34 | 0.11 | 0.47 | **0.06** |
| 14 | low verbal IQ | 0.35 | 0.18 | 0.36 | **0.06** |
| 14 | teacher rated anxious | 0.13 | 0.08 | 0.17 | **0.08** |
| 14 | teacher and self-reported anxiety | 0.31 | 0.28 | 0.06 | **0.10** |
| 14 | teacher rated unpopular | 0.20 | 0.15 | 0.13 | **0.00** |
| 18 | reading ability | 0.12 | 0.05 | 0.23 | **0.11** |

Balance checks: all absolute standardised differences in means should be less than 0.25 for each variable (see Rubin 2001).
